# Nitrogen isotopes provide clues to amino acid metabolism in human colorectal cancer cells

**DOI:** 10.1038/s41598-017-02793-y

**Published:** 2017-05-31

**Authors:** R. V. Krishnamurthy, Yogesh R. Suryawanshi, Karim Essani

**Affiliations:** 1 Department of Geosciences, Western Michigan University, Kalamazoo, MI 49008 USA; 2 Laboratory of Virology, Department of Biological Sciences, Western Michigan University, Kalamazoo, MI 49008-5410 USA

## Abstract

Glutamic acid and alanine make up more than 60 per cent of the total amino acids in the human body. Glutamine is a significant source of energy for cells and also a prime donor of nitrogen in the biosynthesis of many amino acids. Several studies have advocated the role of glutamic acid in cancer therapy. Identification of metabolic signatures in cancer cells will be crucial for advancement of cancer therapies based on the cell’s metabolic state. Stable nitrogen isotope ratios (^15^N/^14^N, δ^15^N) are of particular advantage to understand the metabolic state of cancer cells, since most biochemical reactions involve transfer of nitrogen. In our study, we used the natural abundances of nitrogen isotopes (δ^15^N values) of individual amino acids from human colorectal cancer cell lines to investigate isotope discrimination among amino acids. Significant effects were noticed in the case of glutamic acid, alanine, aspartic acid and proline between cancer and healthy cells. The data suggest that glutamic acid is a nitrogen acceptor while alanine, aspartic acid and proline are nitrogen donors in cancerous cells. One plausible explanation is the transamination of the three acids to produce glutamic acid in cancerous cells.

## Introduction

Application of stable isotope techniques in geochemical research is well known and has a long history^1–4^. In the past decades, extension of these techniques to other fields such as forensics, biomedical and biochemical research has grown rapidly, especially involving ^13^C labelled substrates^5–10^. Clinical pharmacological research takes advantage of the fact that analytical precisions in the detection of stable isotope concentrations have made unparalleled advances. In addition, use of stable isotopes eliminates the need to use radioactive isotopes that might delay the transition of new therapeutics and diagnostics into clinical trials. Applications in biochemical research allow detailed investigation of peptide chemistry, carbohydrate and amino acid metabolism among others. With respect to proteins and amino acids, nitrogen is more attractive since many enzymatic reactions involving amino acids result in transport of nitrogen rather than carbon. These studies are performed using individual amino acids or by looking at isotope variations within a single amino acid (intra molecular) in biochemical and ecological studies^11, 12^.

Cancer cells have been shown to increase the uptake of both glucose and glutamine in order to meet the increased metabolic demand^13–15^. However, cancer cells show a great degree of metabolic heterogeneity in terms of defects in metabolic pathways^16, 17^. Additionally, cancer cell metabolism is also affected by activation of different tumor suppressor genes and oncogenes^18^. Metabolic heterogeneity of cancer cells remains to be a major hurdle in using cancer therapies targeting specific metabolic defects^19, 20^. Stable isotope technique allows a quick analysis of the metabolic status of cancer cells and can be pivotal for development and optimization of cancer therapies targeting specific metabolic pathways.

Natural abundances of ^13^C and ^15^N isotope in human breast cancer biopsy tissues and human breast cancer cell lines have been used to determine an isotopic difference between healthy and breast cancer cells which correlates with the metabolic status^21^. Here, we measured the natural abundances of stable nitrogen isotope ratios in individual amino acids in human colorectal cancer cells, grown *in vitro*, under identical conditions. The objective of our study is to examine if there is any preferential amino acid usage in human colorectal cancer cells versus the non-cancerous human cells. Growing the cells in identical condition and medium was intended to eliminate or minimize substrate bias.

## Results

### Total cell lines or biomass

All the isotope data are provided in Table 1 in the usual δ notation as shown in equation 1

1 $${\delta }^{15}{\rm{N}}\,permil=(\frac{{{\rm{R}}}_{{\rm{S}}{\rm{a}}{\rm{m}}{\rm{p}}{\rm{l}}{\rm{e}}}}{{{\rm{R}}}_{{\rm{S}}{\rm{t}}{\rm{a}}{\rm{n}}{\rm{d}}{\rm{a}}{\rm{r}}{\rm{d}}}}-1)\times {10}^{3},{\rm{w}}{\rm{h}}{\rm{e}}{\rm{r}}{\rm{e}}\,{\rm{R}}={}^{15}{\rm{N}}{/}^{14}{\rm{N}},{\rm{t}}{\rm{h}}{\rm{e}}\,{\rm{S}}{\rm{t}}{\rm{a}}{\rm{n}}{\rm{d}}{\rm{a}}{\rm{r}}{\rm{d}}\,{\rm{i}}{\rm{s}}\,{\rm{A}}{\rm{i}}{\rm{r}}\,{\rm{N}}{\rm{i}}{\rm{t}}{\rm{r}}{\rm{o}}{\rm{g}}{\rm{e}}{\rm{n}}.\,$$

**Table 1:** δ^15^N values of total biomass and individual amino acids representing healthy cells and human colorectal cancer cells.Total biomass represents those which were used for individual amino acid extraction.

Total biomass										
Sample				δ^15^N				Comments		
WI-38				3.48				Healthy cells		
SW1463				−1.15				Colorectal cancer cell		
WiDr				−1.47				Colorectal cancer cell		
HCT116				−1.73				Colorectal cancer cell		
COLO 205				−0.84				Colorectal cancer cell		
**δ** ^**15**^ **N values of Individual Amino Acids**										
(Ile = Isoleucine, Leu = Leucine, Lys = Lysine, Phe = Phenylalanine, Val = Valine, Ala = Alanine, Asx = Aspartic acid, Glx = Glutamic acid, Pro = Proline, Gly = Glycine)										
**Sample**	**Ile**	**Leu**	**Lys**	**Phe**	**Val**	**Ala**	**Asx**	**Glx**	**Pro**	**Gly**
WI-38 (Healthy cells average ± 1σ, n = 4)	5.28 ± 1.6	4.63 ± 1.8	1.24 ± 1.7	3.13 ± 4.7	8.69 ± 1.4	4.59 ± 1.2	5.78 ± 1.4	5.54 ± 1.7	6.61 ± 3.7	4.64 ± 1.5
SW1463	5.38	5.56	5.14	10.3	12	9.13	7.2	−1.6	11.9	7.6
WiDr	1.91	4.16	2.1	5.24	5.93	11.8	9.8	−7.8	15	3.9
HCT116	3.39	4.91	3.23	7.67	7.43	15.1	8.2	−3.5	12.7	7.5
COLO 205	3.55	4.36	0.42	6.34	8.89	10.86	8.66	−2.47	11.87	12.65
Cancerous Cells, average ± 1σ n = 4	3.56 ± 1.4	4.75 ± 0.6	2.72 ± 1.9	7.4 ± 2.2	8.57 ± 2.6	11.73 ± 2.5	8.49 ± 1	−3.83 ± 2.3	12.9 ± 1.5	7.9 ± 3.6

Individual amino acid nitrogen isotope analysis: Individual amino acids (free and protein bound) were prepared from cell lines using the chloroformate extraction technique (UC Davis Stable Isotope Facility)^22^. A recent study compares the chloroformate extraction technique favourably with other standard extraction techniques^23^. Analytical precision of δ^15^N measurements is reported to be 1.5‰. It should be noted that during acid hydrolysis of samples to separate individual amino acids for isotope measurements, glutamine (Gln) and glutamic acid (Glu) are both converted to the acid form glutamic acid and glutamate, generally represented as Glu^24^. Therefore, it is sometimes common practice to denote Glu as Glx. Similarly, aspartic acid is converted to aspartate and the acid is represented as Asx. In either case it has been reported that nitrogen isotope fractionation does not take place during hydrolysis^11, 24^. Note that isotope values of all extractable amino acids have been included.

There are reports of the effects of hypoxia on tumor cells under hypoxic conditions where cancer cells may switch to alternative amino acid sources^25^. Usually tumor cells at the core of tumor mass face the hypoxic conditions due to rapid utilization of energy sources owing to the extensive rate of proliferation. However, in our study the cells were grown *in vitro*, which is unlikely to put cells under hypoxic stress and lead to stress-induced switch of amino acid usage.

The total biomass δ^15^N values of all the cancerous cells are negative compared to the healthy cells. The total biomass represents those that were used for later amino acid extraction. The isotopic trend is similar to what has been reported in a previous study on breast cancer tissues^21^. This observation, although systematic has limited implications. This points out, as shown in previous studies, to the need to carry out compound specific isotope analysis in pharmacological, ecological and biochemical research. The main focus in this study is therefore on compound specific isotope ratios, namely those of individual amino acids.

**Figure 1:**
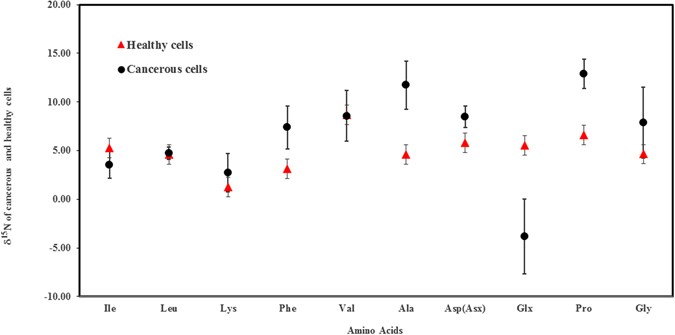
δ^15^N values of amino acids extracted from human colorectal cancerous and healthy cells. The error bars are based on the average of four samples prepared independently. The amino acids are: Isoleucine (Ile), Leucine (Leu), Lysine (Lys), Phenylalanine (Phe), Valine (Val), Alanine (Ala), and Aspartic acid (Asp/Asx), Glutmaic acid (Glx), Proline (Pro) and Glycine (Gly). The δ^15^N values of amino acids glutamic acid, alanine, aspartic acid and proline showed statistically significant differences between the healthy and cancerous human cells.

Comparative analysis showed that the average δ^15^N values of four amino acids, namely glutamic acid (p = 0.001; degree of freedom = 3), alanine (p = 0.002; degree of freedom = 3), aspartic acid (p = 0.021; degree of freedom = 3) and proline (p = 0.020; degree of freedom = 3), were significantly different between human colorectal cancer and healthy cells. Glutamic acid, with an average δ^15^N value of about −4‰ is depleted, while alanine, aspartic acid and proline are enriched in δ^15^N in cancerous cells compared to the healthy cells (Fig. 1).

Since both healthy cells and cancerous cells were grown under identical conditions using the same media that had added glutamine as a source of energy and other sources of essential amino acids, and the objective of this study is to compare nitrogen isotope fractionation between healthy and cancerous cells, it is therefore more pertinent to compare fractionation effects between the cancerous cells and the healthy cells. We can make use of the “per mil fractionation” defined as in equation 2:

2 $${10}^{3}\,\mathrm{ln}\,{\alpha }_{B}^{A}\approx {\delta }_{A}-{\delta }_{B}={{\rm{\Delta }}}_{B}^{A}$$

where α is the fractionation factor between A, the amino acids in cancerous, and B, those in healthy cells respectively.

The calculated Δ values (per mil fractionation) between the cancerous and healthy human cells for four amino acids that showed statistically significant δ^15^N values between healthy and cancerous cells are shown in Table 2.

**Table 2:** Per mil fractionation (Δ^15^N) difference between cancerous (A) and healthy (B) cells for Glutamic acid, Alanine, Aspartic acid and Proline.

Acids	Δ^15^N‰ (δ^15^N_cancerous_ − δ^15^N_healthy_)
Glutamic acid	−9.37
Alanine	7.14
Aspartic acid	2.71
Proline	6.28

## Discussion

As stated above there are striking differences in fractionation in glutamic acid compared to alanine, aspartic acid and proline. It is well known that biochemical reactions mediated by enzymes are accompanied by a Kinetic Isotope Effect (KIE) such that the lighter isotopes are preferentially removed leaving the substrate enriched in the heavier isotope. This is due to the fact that less energy is required to break the bonds made up of the lighter isotopes. The more efficient this process is, the heavier the “reservoir” will become. If the entire reservoir is used up, there is no net fractionation.

Comparison of δ^15^N values of healthy and cancerous cells indicate that glutamic acid is a nitrogen acceptor whereas alanine, aspartic acid and proline are nitrogen donors. Glutamine has been implicated in glucose production (energy source) via the gluconeogenesis pathway^26^. A detailed study of glutamine metabolism also invokes deamination of glutamine as an important step in energy production^27^. Understanding the energy source indeed requires 13-C labeled investigations using Isotope Ratio Mass Spectrometry or NMR spectroscopy^28, 29^. In terms of nitrogen isotope fractionation, the deamination is the most significant step since further reactions leading to gluconeogenesis do not involve nitrogen.

Glutamic acid is considered a prime nitrogen donor in the synthesis of many amino acids in a direct or indirect manner. Synthesis of other amino acids involves deamination of glutamic acid, accompanied by KIE such that the product is depleted in δ^15^N and the substrate glutamic acid is enriched in δ^15^N. This was demonstrated in a pioneering study where it was shown that conversion of glutamic acid to aspartic acid resulted in the aspartic acid being depleted in δ^15^N, in conformity with known biochemical KIE fractionation^30^. Similar observations were made, but with lesser isotope effect in a study of fractionation when glutamic acid was converted to aspartic acid using reagent chemistry^31^. In another recent study involving bacteria cultured in aerobic and anaerobic conditions, an enrichment of δ^15^N in glutamic acid was again shown to be in agreement with expected fractionation^11^. In all these studies the glutamic acid was seen to be a nitrogen donor resulting in the expected KIE. This study on the other hand suggests that glutamic acid in human colorectal cancer cells is an acceptor of the amino group. The isotopic support is shown in Table 3 where the Δ^15^N values between glutamic acid and the other three amino acids (alanine, aspartic acid and proline) in human colorectal cancer cells show large differences. Such differences in the healthy cells are too small and within analytical errors. Moreover the differences in Δ^15^N between cancerous and healthy cells were statistically significant (p < 0.05).

**Table 3:** Per mil fractionation (Δ^15^N) between Glutamic acid and Alanine, Glutamic acid and Aspartic acid and Glutamic acid Proline in cancerous and healthy human cells.

Acids	Δ^15^N‰ (Cancerous cells)	Δ^15^N‰ (Healthy cells)
(δ^15^N_Glu_ − δ^15^N_Ala_)	−15.6	0.95
(δ^15^N_Glu_ − δ^15^N_Asp_)	−12.32	0.83
(δ^15^N_Glu_ − δ^15^N_Pro_)	−16.72	−0.9

**Figure 2:**
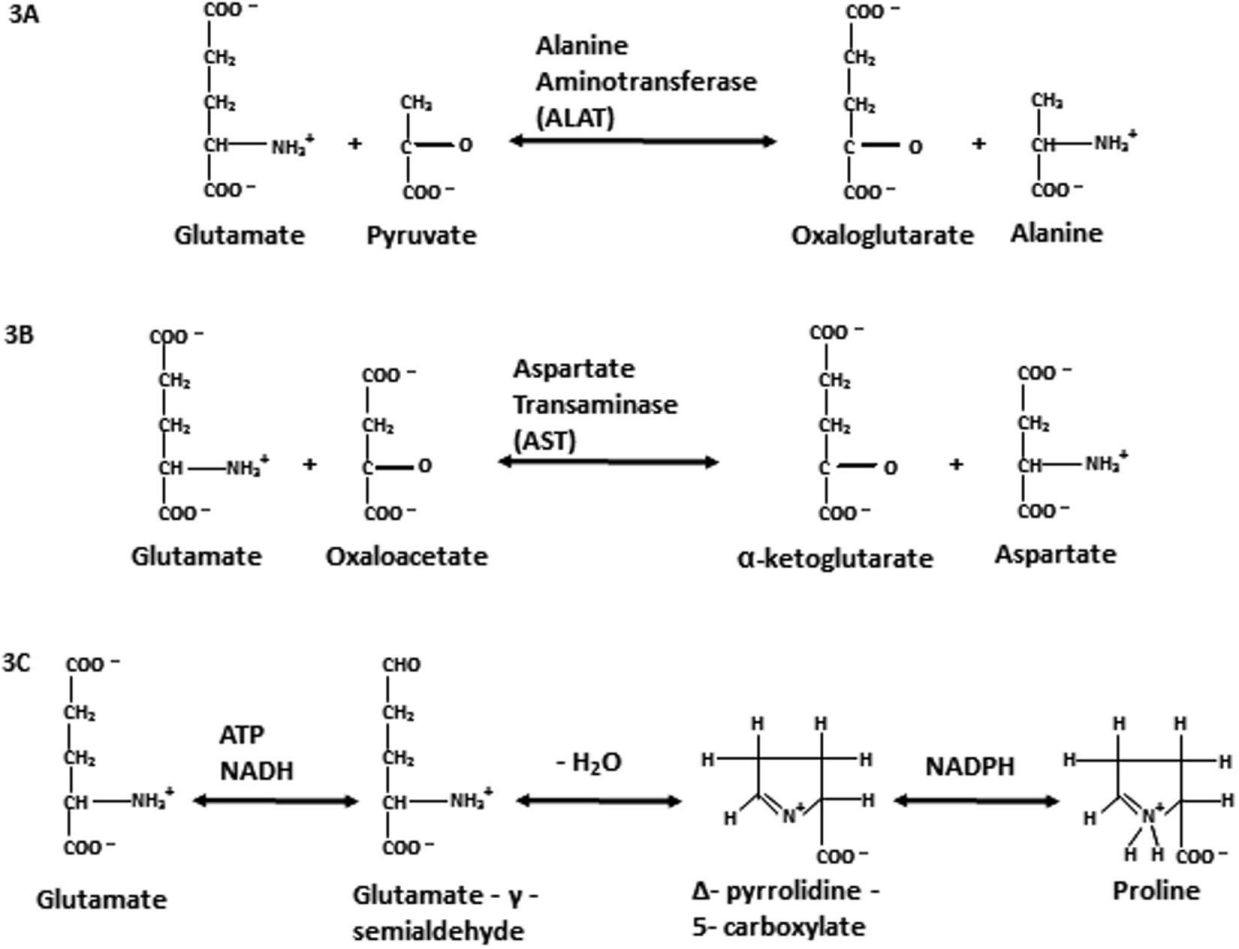
Schematic showing reactions involving interconversion between glutamine and alanine (**A**), aspartic acid (**B**) and proline (**C**), respectively. ATP = Adenosine triphosphate; NADH = Nicotinamide adenine dinucleotide; NADPH = Nicotinamide adenine dinucleotide phosphate.

One reasonable approach to explain the behavior of glutamic acid and the other three acids (alanine, aspartic acid and proline) in cancer cells as the amine group acceptor and donors, respectively, is to consider the conversion of alanine, aspartic acid and proline to glutamic acid. Conversion of glutamate to alanine, aspartate and proline has been recognized. The suggested pathways are shown in a simplified schematic in Fig. 2A–C involving Adenosine triphosphate (ATP), Nicotinamide adenine dinucleotide (NADH) and Nicotinamide adenine dinucleotide phosphate (NADPH). If these reactions are reversible in the cancerous cells, the glutamic acid will result in depleted δ^15^N.

To summarize, our study showed that isotope analysis can be a quick method to analyze the metabolic status of cancer cells. The colorectal cancer cell lines used in our study showed that glutamic acid is a nitrogen acceptor. This observation assumes significance considering that there are recent studies that advocate glutamine in cancer therapy^32–37^. We propose that isotope analysis can be used effectively to determine the metabolic status of a cancer type which may be helpful in determining the optimum treatment strategy.

## Materials and Methods

### Preparation of cell samples for isotope analysis

Human fetal lung fibroblasts (WI38) [ATCC-CCL75], and human colorectal cancer cells WiDr [ATCC-CCL218], HCT 116 [ATCC-CCL247], COLO 205 [ATCC-CCL222] and SW1463 [ATCC-CCL234] were purchased from the American Type Culture Collection. All cells were grown in monolayers using 75 cm^2^ tissue culture flasks (10^7^ cells/flask) that were incubated at 37 °C in a 5% CO2 atmosphere. All cell lines were propagated in complete growth medium consisting of Dulbecco’s Modified Eagle’s Medium (Gibco/Life Technologies) supplemented with 10% (vol/vol) fetal bovine serum (FBS) (Atlanta Biologicals), 2 mM L-glutamine (Sigma-Aldrich) and, 50 I.U./ml Penicillin and 50 μg/ml streptomycin (AMRESCO). All cells were cultured a day before, so they reach a confluency on the next day, when cells were harvested for the isotope analysis. All the cell lines were cultured and treated in an identical manner.

### Isotope analysis

Isotope measurements of bulk samples were performed at the Michigan State University and those of individual amino acids at the Stable Isotope Facility of University of California Davis. Acid hydrolysis using 6 M HCl was used to release individual amino acids and a modified chloroformate derivatization was used to prepare samples for IRMS in a Delta V Advantage isotope ratio mass spectrometer coupled to a GC Combustion Interface III (Thermo Electron, Bremen, Germany). NIST Standard Reference Materials were used for data analysis and corrections. Analytical precision of δ^15^N measurement is better than 1.5‰. Detailed protocol of isotope analysis can be found in ref. 22. Comparison of this method technique with other commonly used method is reported in ref. 23.

### Statistics

To assess the metabolic differences between human colorectal cancer cells and healthy cells, pairwise comparison was carried out (using student T test) between the δ^15^N values of each amino acid in human colorectal cancer cells (n = 4) with the δ^15^N values for the same amino acid in healthy cells (n = 4), with a significance level of p < 0.05.
